# Use of a Free Vascularized Medial Femoral Condyle Flap for Revision Surgery in a Pediatric Patient with Congenital Pseudarthrosis of the Clavicle

**DOI:** 10.1155/2020/8872934

**Published:** 2020-06-29

**Authors:** Atsuro Murai, Kaoru Tada, Mika Nakada, Masashi Matsuta, Katsuhiro Hayashi, Hiroyuki Tsuchiya

**Affiliations:** Department of Orthopaedic Surgery, Graduate School of Medical Science Kanazawa University, 13-1 Takaramachi, Kanazawa, Ishikawa 920-1302, Japan

## Abstract

The most common surgical treatment for congenital pseudarthrosis of the clavicle (CPC) is resection of the pseudarthrosis, placement of an autologous bone graft, and Kirschner wire or plate fixation. However, in some cases, bone fusion cannot be achieved at the first surgery, and an additional surgery is required. We present a case report of a boy with a right CPC who failed radiographic bone union after the first surgery. He subsequently underwent revision surgery with resection of the pseudarthrosis, plate fixation, and establishment of a vascularized medial femoral condyle (MFC) flap to ensure bone union. Three months after the revision surgery, a radiographic bone union was achieved, and no symptoms were observed for one year after the operation. There have been no previous reports of the use of a vascularized MFC flap as a treatment for CPC. We believe that this technique effectively ensures bone union during revision surgery for CPC.

## 1. Introduction

Congenital pseudarthrosis of the clavicle (CPC) is a rare condition that occurs at birth and is often diagnosed in late childhood when the clavicle deformity becomes obvious [1]. Conservative treatment is usually performed when there are no symptoms; however, surgical treatment is indicated when pain, functional impairment, cosmetic problems, and neurovascular structure compression syndrome develops [2–4].

The most common surgical treatment is the resection of the pseudarthrosis, placement of a free iliac bone graft, and Kirschner wire (KW) or plate fixation [5–9]. However, in some cases, bone fusion cannot be achieved during the first surgery, and an additional surgery is required [6–9]. Although there are some reports of revision surgery, except for one report using vascularized fibular graft [10], most of them only repeated resection of the pseudarthrosis, placement of a free bone graft, and internal fixation.

We report a case of CPC that required revision surgery after failed first surgery with resection of the pseudarthrosis, placement of a free iliac bone graft, and plate fixation. We performed a second surgery, which used a vascularized medial femoral condyle (MFC) flap, in addition to resection of the pseudarthrosis and plate fixation to ensure bone union.

## 2. Case Presentation

**Figure 1:**
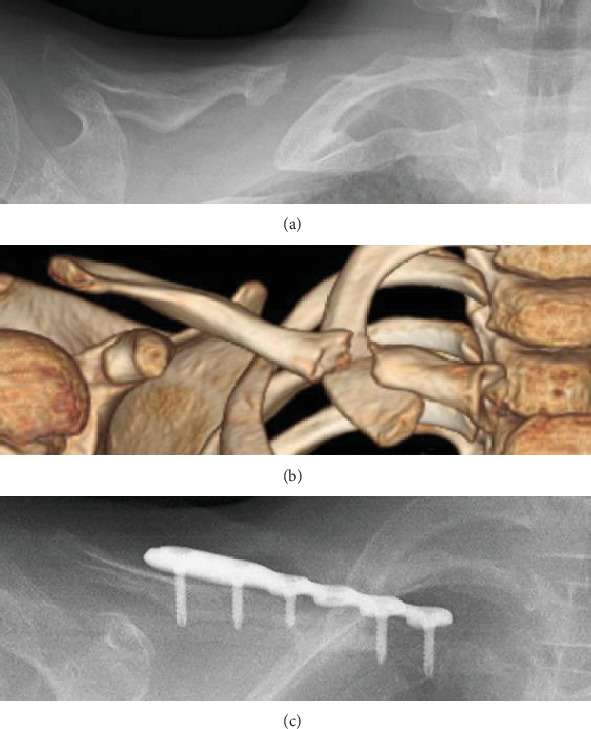
(a, b) 10 mm sized defect of the middle third of the right clavicle. (c) Initial surgery with resection of the pseudarthrosis, placement of an autologous iliac bone graft, and plate fixation.

A 5-year old boy visited a hospital for right clavicle pain after slight trauma to his right shoulder. Radiography revealed a right clavicle fracture, and he was treated with a cast. However, bone nonunion persisted even after 3 months, necessitating referral to our hospital for further evaluation. Evaluation revealed portal hypertension and familial exudative vitreoretinopathy. Radiography and computed tomography revealed a 10 mm sized defect of the middle third of the right clavicle (Figures 1(a) and 1(b)), and the patient was diagnosed with CPC. His pain had reduced at the time of presentation to our hospital; therefore, we did not perform internal fixation.

The patient returned 5 years later (at 10 years of age) with worsened right clavicle pain while playing kendo and pain induced-restricted range of motion of his right shoulder. His pain was attributed to CPC, and we performed surgery with pseudoarthrosis resection, placement of an autologous iliac bone graft, and plate fixation (Zimmer-Biomet A.L.P.S. distal fibula plate with six holes) (Figure 1(c)).

**Figure 2:**
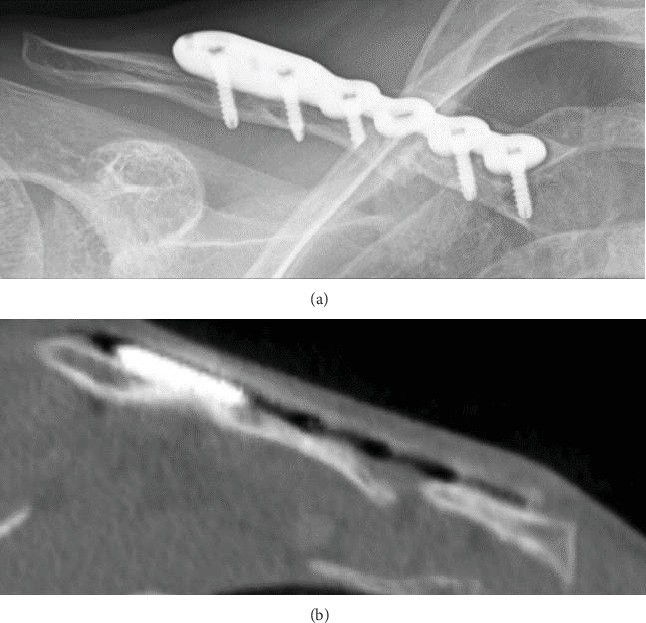
(a, b) Radiographs and computed tomography revealed nonunion and implant loosening one year after the initial surgery.

**Figure 3:**
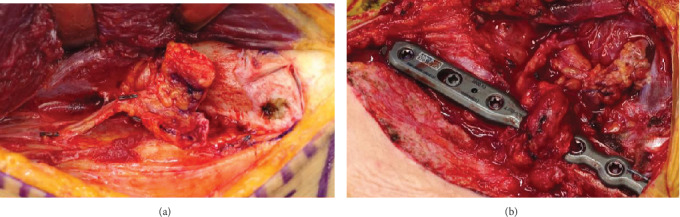
(a) A thin periosteum MFC flap was harvested. (b) MFC flap is wrapped around the clavicle.

**Figure 4:**
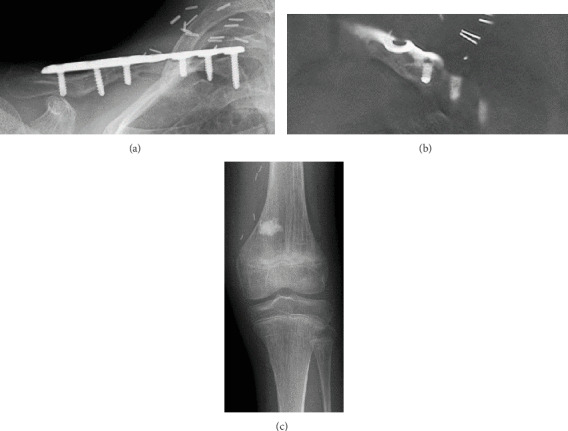
(a, b) Radiographic and tomographic bone fusion was achieved three months after the second operation. (c) The graft could be harvested without damaging the epiphyseal line.

Unfortunately, radiographic bone nonunion persisted, and we observed implant loosening one year later (Figure 2). He was subsequently referred for revision surgery with pseudoarthrosis resection, plate replacement, and application of a vascularized medial femoral condyle flap. The patient was placed under general anesthesia. His previous incision was used to remove the plate. We noted fibrous tissue between the gap, and we debrided the pseudarthrosis. We fixed the clavicle with a new locking plate (Zimmer-Biomet ALPS distal fibula plate with seven holes). Using the descending genicular artery and vein as a vascular pedicle from the left MFC, a thin periosteal 20 × 10 mm MFC flap was harvested (Figure 3(a)). The bony defect between the clavicle was 1 cm, and the area between the defect was packed with cancellous bone from the MFC and wrapped around the clavicle with a periosteal MFC graft (Figure 3(b)). Microvascular anastomoses were performed on the transverse cervical artery and vein. The collected part of the femoral cancellous bone was filled with beta-tricalcium phosphate (*β*-TCP). No postoperative external fixation was performed. Three months after the revision surgery, radiographic bone fusion was achieved (Figure 4(a)), and no symptoms such as pain or limited shoulder joint range of motion were observed for one year after the operation.

## 3. Discussion

Although there are many reports of CPC, indications for surgical treatment are still controversial. Some authors suggest conservative treatment if there are no symptoms [2, 11], and others actively recommend surgical treatment, regardless of the presence or absence of symptoms [12, 15].

Surgical treatment is recommended because of pain, neurological disorders such as thoracic outlet syndrome, or cosmetic issues. Shalom reported on a 45-year-old asymptomatic patient with CPC and recommended conservative management if there are no symptoms [11]. On the other hand, even if there are no symptoms, some authors recommend early surgical treatment because the operation is easier before the gap enlarges with age [3, 6, 12]. However, Giwnewer et al. found that no clinical references were observed between patients who underwent earlier and late-stage operations [13].

Surgery for CPC typically involves resection of the pseudarthrosis, placement of the free iliac bone graft, and KW or plate fixation. Grogan et al. reported that internal fixation should not be used in patients younger than 3 years of age [14]; however, Kim et al. reported on nine infants younger than 18 months who underwent surgery without internal fixation; unfortunately, 43% of these patients could not achieve bony union [8].

Regarding internal fixation materials, some authors have reported that KW fixation is preferred because this method avoids the risk of plate breakage and infection [6, 7]. Other authors prefer plate fixation because this method is associated with a lower risk of false joints [8, 9, 15]. We used a locking plate to obtain strong fixation.

Several authors report that bone fusion was not obtained after the initial operation. Among the cases where bone fusion was not achieved after KW fixation, bone fusion was achieved by plate fixation [9]. In cases where no bone fusion was achieved after plate fixation, one study reported that bone fusion was achieved by reimplanting the bone and refixing with the plate [8]. However, since there is no additional procedure, there is still concern about whether or not the second surgery ensures bone union.

Vascularized bone grafting is considered an effective treatment for intractable pseudarthrosis. However, we only found one report that used a vascularized bone graft for CPC surgery. A vascularized fibular graft was used for revision surgery after a failed initial surgery that used plate fixation [10].

In our case, the defect required the use of a fibular graft. There is a risk of ankle valgus when using a fibular graft. Therefore, we used vascularized bone grafts from MFCs, which have few complications at the donor site and good knee range of motion after surgery [16, 17].

The use of a vascularized MFC flap was first reported by Sakai et al. in 1991 [18]. This flap is relatively easy to harvest and convenient to shape and can be used for various bones such as the femur, tibia, clavicle, distal radius, scaphoid, and talus [19]. It is based on the articular branch of the descending genicular artery and consists of a periosteum with a thin layer of the outer cortical bone.

Several authors have reported good results using a vascularized MFC flap for nonunion of the clavicle in adults [20–22]. The benefit of a free vascularized bone graft from the MFC to the clavicle is that it can be harvested as periosteum with a thin bone to cover the pseudarthrosis. This type of graft also reduces skin stress when the superficial soft tissue is thin like the clavicle [21].

Regarding graft size, Yamamoto et al. reported that the descending genicular artery had average distances proximal to the articular surface of 13.7 cm [23]. Iorio et al. also reported that the descending genicular artery was proximal to the articular surface of 13.7 cm, which is 29% of the total femur length [24]. Although we can harvest an MFC flap greater than 10 cm in length, based on the recommendations set forth by these reports, Endara et al. reported that harvesting more than 7 cm is a risk factor for iatrogenic fractures [25]. Haines et al. reported that harvesting a 6 cm graft resulted in an iatrogenic femoral fracture [26]. Hamada et al. explain that, to prevent fractures, the harvested graft size should be up to 3 × 3 cm [22].

MFC flaps are rarely used in children, and it is difficult to determine the available size of grafts in children due to differences in femoral length [27]. We believe that it is important to determine the size of the graft from each femur length, considering that 29% of the distal joint surface can be used. It is also important not to injure the epiphyseal line in patients where the epiphyseal line remains. In our case, we used fluoroscopy and carefully harvested thin periosteal flap not to damage the epiphysis. Although it might have been better to use vascularized block bone, we were afraid that growth failure or femoral fracture to use the block bone and used periosteal thin flap. As a result, the graft could be harvested without damaging the epiphyseal line (Figure 4(b)), and there are no obvious complications related to the donor site at present; however, careful observation is required.

Previously, there were no reports of the use of a vascularized MFC flap as a treatment for CPC; however, our case experienced a good postoperative course. Although this MFC flap is not necessarily suitable as an initial surgery, we believe that this technique is an effective method to ensure bone union during revision surgery for CPC.

## Data Availability

The [DATA TYPE] data used to support the findings of this study are included within the article.
